# Impaired psychosocial functioning and emotion regulation difficulties in eating disorders: a cross-sectional study

**DOI:** 10.1186/s40337-026-01803-7

**Published:** 2026-10-22

**Authors:** Louise Bergman, Mia Ramklint, Vendela Zetterqvist, Martina Isaksson

**Affiliations:** Department of Medical Sciences, Child and Adolescent Psychiatry, Uppsala University, Entrance 10, Floor 3B, Uppsala, SE-751 85 Sweden

**Keywords:** Eating disorders, Anorexia nervosa, Bulimia nervosa, Psychosocial functioning, Emotion regulation

## Abstract

**Background:**

Eating disorders (EDs) are severe mental disorders marked by disturbed eating and body-related concerns, leading to significant distress and impairment. However, psychosocial functioning in ED diagnoses remains understudied. Emotion dysregulation is a central feature of EDs, linked to their development and severity, and is known to predict functional impairment in other mental disorders. However, its role in psychosocial functioning in EDs remains unclear. This cross-sectional study aimed to compare psychosocial functioning across ED diagnoses, identify subgroups based on emotion dysregulation, and examine the association between emotion regulation and psychosocial functioning while controlling for key clinical and demographic factors.

**Methods:**

In total, n = 193 women were recruited from a specialised ED clinic. All were assessed with diagnostic interview and self-report questionnaires. The questionnaires measured psychosocial functioning, emotion dysregulation, depression, anxiety and non-suicidal self-injury (NSSI). Psychosocial functioning was compared across five ED groups: anorexia nervosa restrictive type (ANr) (n = 30), anorexia nervosa binge eating/purging type (ANbp) (n = 32), bulimia nervosa (BN) (n = 74), other specified feeding or eating disorders atypical anorexia nervosa (OSFED AN) (n = 25), and OSFED bulimia nervosa/purging disorder (OSFED BN) (n = 32). Emotion regulation sub-groups were extracted through latent class analysis (LCA). A hierarchical linear regression examined the impact of emotion regulation on psychosocial functioning, controlling for age, depression, and anxiety.

**Results:**

Patients with ANr and OSFED BN showed the lowest levels of psychosocial impairment, whereas those with BN and OSFED AN had the most impaired functioning. A latent class analysis revealed three distinct emotion dysregulation profiles; low, intermediate and high emotion dysregulation. These were significantly associated with eating pathology, depression, anxiety, NSSI, and compensatory behaviours. Emotion dysregulation was not a significant predictor of psychosocial functioning when controlling for age and symptoms of depression and anxiety; instead, depression and ED diagnosis accounted for significant variance.

**Conclusion:**

Psychosocial functioning differed across EDs but was not explained by emotion dysregulation. Three emotion dysregulation profiles were found that were associated with symptom severity.

**Plain english summary:**

In this study, we examined how women with different eating disorders function in daily life and whether they differ in how they manage their emotions. We also examined whether difficulties with managing emotions were related to everyday functioning. We collected data from 193 women seeking care for an eating disorder at Uppsala University Hospital. Participants completed questionnaires measuring daily functioning, difficulties with managing emotions, depression, anxiety, and self-harm. We found that women with bulimia nervosa and atypical anorexia nervosa reported greater difficulties in daily functioning than women with other eating disorders. We also identified three groups based on difficulties managing emotions: one with few difficulties, one with moderate difficulties, and one with severe difficulties, such as difficulty accepting negative emotions or controlling impulsive behaviour. Women with greater difficulties managing their emotions had more severe eating disorder symptoms, higher levels of depression and anxiety, and more frequent self-harm. However, they reported less use of laxatives and diuretics than women with fewer difficulties managing their emotions. In addition, after taking depression and anxiety into account, difficulties managing emotions were no longer linked to daily functioning.

**Supplementary information:**

The online version contains supplementary material available at https://doi.org/10.1186/s40337-026-01803-7.

Eating disorders (EDs) are characterised by unhealthy behaviours and attitudes towards food, eating, body weight, and body shape. Symptoms include both cognitive (e.g. weight concern) and behavioural (e.g. self-induced vomiting) presentations [1]. EDs often co-occur with other mental disorders, particularly mood and anxiety disorders [2, 3]. Further, they are severe disorders often having detrimental impact on the individual’s wellbeing such as reducing quality of life [4], increasing mortality rates (including suicide) [5, 6], and impairing physical health (e.g. cardiovascular, gastrointestinal, dental, skeletal, reproductive and metabolic complications) [2]. Additionally, EDs may impair psychosocial functioning [7, 8].

Psychosocial functioning refers to the ability to fulfil tasks, roles and requirements in the psychological, social and occupational domain 9. It is an important aspect in diagnostic considerations, prognosis and patient prioritisation [10]. Assessing psychosocial functioning alongside symptom burden is important, as functional impairment does not necessarily correspond directly to symptom severity [11]. For example, some individuals with high PTSD symptoms report no functional impairment [12]. To specifically capture the functional impairment of EDs, the Clinical Impairment Assessment (CIA) self-report scale was developed. It measures impairment in the personal, cognitive and social life domains [13]. However, findings regarding psychosocial functioning across different ED diagnoses remain inconsistent. Some studies have found no differences between diagnoses [14–16]. Others have reported differences in threshold and subthreshold EDs, e.g. higher levels of psychosocial impairment among individuals with Anorexia Nervosa (AN) and Bulimia Nervosa (BN) compared with those with Eating Disorders Not Otherwise Specified (EDNOS) 17. Although ED symptomatology is known to be associated with impaired psychosocial functioning, less is known about the factors associated with poorer psychosocial functioning among individuals with EDs. Difficulties with emotion regulation may be one such factor.

Emotion regulation refers to the ability to modulate emotional arousal and encompasses awareness, understanding and acceptance of emotions, as well as the ability to act in accordance with one’s goals regardless of one’s emotional state [18]. Difficulties in emotion regulation have been identified as both a core factor in the development and maintenance of EDs (e.g., Oldershaw et al., [19], Racine, [20] and an important target for intervention [21–23]. Emotion dysregulation has also been associated with greater ED symptom severity [23], with individuals with EDs showing significantly higher levels of emotion dysregulation than healthy controls (e.g., [24], [19]). Further, it has also been suggested that there are subgroups of varying emotion regulation deficits in the ED population [25]. These findings highlight the importance of emotion regulation in understanding ED psychopathology.

While difficulties with emotion regulation have been linked to ED symptomology, less is known about their association with psychosocial functioning specifically. Research in other psychiatric populations suggests that difficulties with emotion regulation may also be associated with poorer psychosocial functioning. Emotion dysregulation has been associated with poorer psychosocial functioning in a series of psychiatric disorders, such as bipolar disorder [26], PTSD [27] ADHD and autism [28, 29]. However, whether difficulties with emotion regulation are similarly associated with psychosocial functioning among individuals with EDs remains unclear.

In the current study, we aimed to (1) investigate psychosocial impairment across different ED diagnoses, (2) determine whether individuals with eating disorders form distinct subgroups based on emotion dysregulation, and (3) examine the association between emotion dysregulation and psychosocial impairment while controlling for relevant demographic and clinical variables. Given the mixed findings in previous research, the first aim was exploratory. For the second aim, we hypothesised that distinct subgroups based on emotion dysregulation would be identified. For the third aim, we hypothesised that emotion dysregulation would be associated with higher levels of psychosocial impairment.

## Methods

### Procedure

The study used an observational cross-sectional design. Participants were recruited during their first routine assessment at the eating disorder clinic at Uppsala University Hospital, from October 2014 to December 2019. They received verbal and written information about the study. Participants provided written informed consent before filling out the questionnaires described below. No compensation was provided for participation.

All participants underwent a diagnostic assessment using the eating disorder examination interview (EDE) and BMI. The EDE is a semi-structured interview designed to assess of ED psychopathology, primarily focusing on the preceding 28 days [30]. Version 12.0D [30] was used in the current study. Fifteen psychologists were trained to administer the EDE (see [31]; for further details of the procedure), and demonstrated complete diagnostic agreement. Diagnoses were initially set according to DSM-IV-TR^9^, and subsequently recoded according to DSM 5 criteria^32^ . Diagnoses were recoded according to DSM-5 criteria, as data collection began before the introduction of DSM 5 at the clinic. Thus, patients who did not receive an AN diagnosis according to DSM-IV-TR, due to 17.5 kg/m^2^ < BMI < 18.5 kg/m^2^ and absence of amenorrhea were included in the AN groups after recoding to DSM-5. Patients fulfilling all AN criteria except from BMI criterion (BMI < 18.5 kg/m^2^) were classified as having Atypical AN. DMS 5 requires binge eating and compensatory behaviours at least once per week, compared with at least twice per week according to DSM-IV-TR. Patients who met the revised DSM-5 frequency criterion were therefore classified as having BN.

### Participants

A total of 193 patients with EDs participated in the study. Inclusion criteria were an age of $\:\ge\:$ 18 years and a current ED diagnosis. Participants had one of six ED diagnoses or subtypes, grouped into five diagnostic categories: Anorexia Nervosa, restricting type (ANr; *n* = 30), Anorexia Nervosa, binge eating/purging type (ANbp; *n* = 32), Bulimia Nervosa (BN; *n* = 74), Other Specified Feeding or Eating Disorders Atypical Anorexia Nervosa (patients with significant weight loss, but whose weight is still within or above normal range) (OSFED AN; *n* = 25) and Other Specified Eating and Feeding Disorders Bulimia Nervosa or Purging Disorder (OSFED BN; *n* = 32). The two OSFED subtypes were grouped into a single category because of their shared clinical characteristics, including the presence of compensatory behaviours (e.g. self-induced vomiting). This grouping was also used to ensure sufficient sample sizes for comparisons across diagnostic categories.

**Fig. 1:**
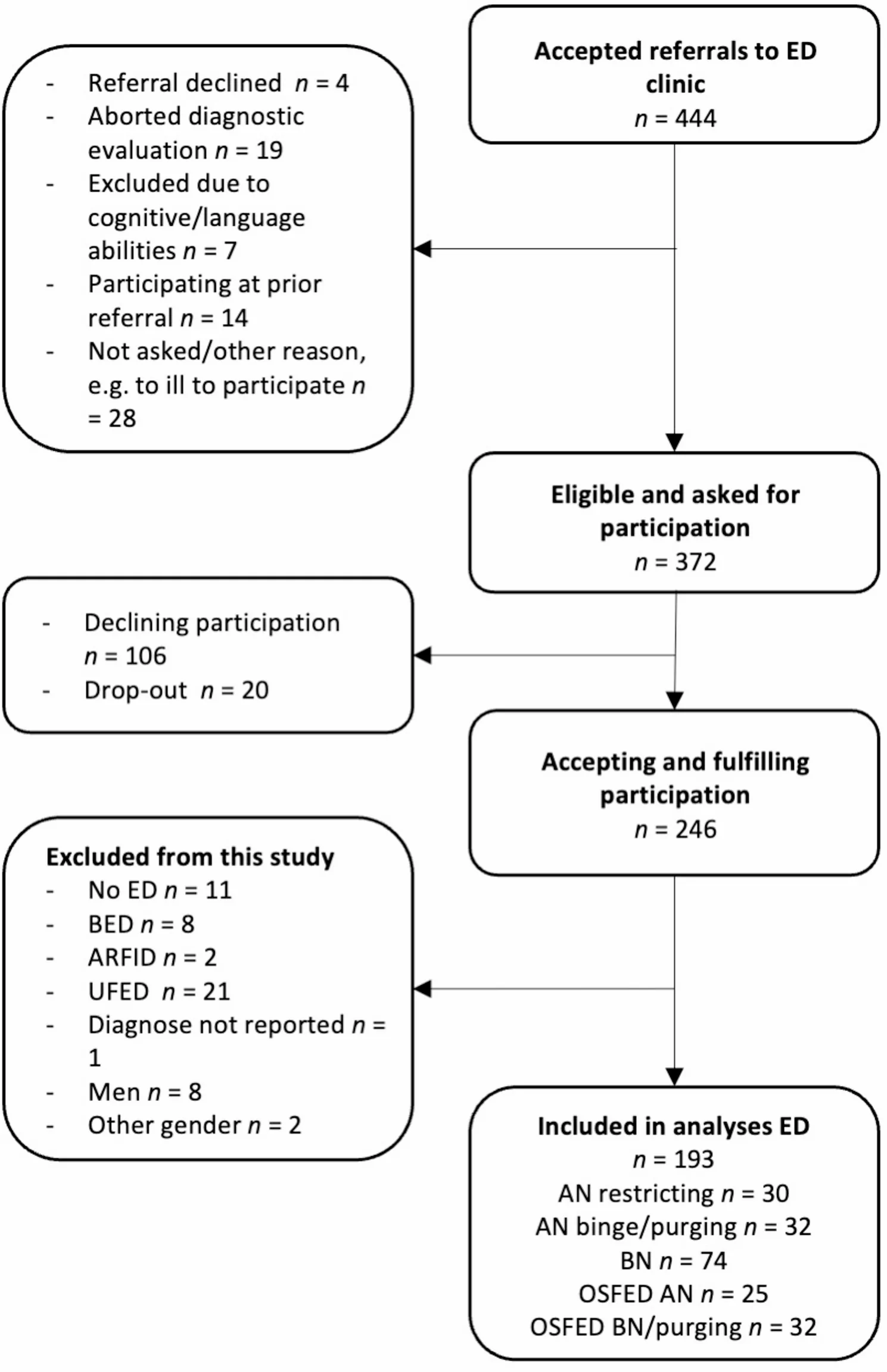
Flowchart of participant recruitment

Exclusion criteria were an inability to complete the questionnaires due to cognitive or language difficulties or e.g. being too ill in their ED, as well as a diagnosis of Binge Eating Disorder, Unspecified Eating or Feeding Disorder, or Avoidant/Restrictice Food Intake Disorder. These patient groups were excluded as they were typically referred to primary care or other clinics rather than to the specialist clinic for EDs. In addition, male and non-binary patients were excluded due to the small number of participants, which limited the ability to control for them in the analyses and prevented meaningful conclusions from being drawn for these populations. The recruitment flowchart is presented in Fig. 1, and participant demographics in Table 1.

**Table 1:** Demographic characteristics of study participants *n* = 193

Mean age (SD)	*n*	%
	25.4 (6.8)	
Mean BMI (*SD*)^a^	21.4 (4.8)	
Marital status^b^		
Partner	65	33.7
Single	117	60.6
Other (e.g. living with parents)	8	4.1
Highest level of education^c^		
Primary school	28	14.5
Secondary school	93	48.2
Higher education	71	36.8
Main occupation^d^		
Paid work	68	35.3
Student	79	40.9
Unemployed	4	2.1
Sick leave	36	18.7
Other (e.g. parental leave, internship)	4	2.0

Note. a: *n* = 4 missing, b: *n* = 3 missing, c: *n* = 1 missing, d: *n* = 2 missing

### Materials

#### Self-report scales

##### Clinical impairment assessment questionnaire (CIA)

The Clinical Impairment Assessment questionnaire (CIA) [13] assesses the degree of psychosocial impairment due to eating disorder symptoms during the past 28 days. It covers several domains in life: personal, cognitive functioning and social. The 16-item self-report scale is rated on a 4-point Likert scale ranging from 0 (“Not at all”) to 3 (“A lot”). Items are summed up to yield a global score ranging from 0 to 48, with a higher score being indicative of a more severe impairment. A global score of ≥ 16 is indicative of a clinically significant impairment due to an ED [33]. The scale has demonstrated an excellent internal consistency (α = 0.97), acceptable test-retest reliability, and satisfactory construct and discriminant validity [13]. Swedish norms are also available 17. The Swedish version was used and demonstrated excellent internal consistency (α = 0.90)

##### Difficulties in emotion regulation scale (brief version) (DERS-16)

Difficulties in emotion regulation scale (DERS) [18] measures emotion regulation difficulties across five dimensions. These dimensions are nonacceptance of negative emotions, inability to engage in goal-directed behaviours during distress, difficulties controlling impulsive behaviours during distress, limited access to emotion regulation strategies perceived as effective, and lack of emotional clarity. The scale is answered on a five-point Likert scale ranging from 1 (“Almost never”) to 5 (“Almost always”). The brief Swedish 16 item version was used (DERS-16) [34]. The scale is answered on a five-point Likert scale ranging from 1 (“Almost never”) to 5 (“Almost always”). Items are summed up to a total score ranging from 16 to 80, higher scores reflecting greater levels of emotion dysregulation. The scale has shown satisfactory psychometric properties; excellent internal consistency (*α* = 0.92), good test-retest reliability (*ρI* = 0.85; *p* < .001), and satisfactory convergent validity [34]. The internal consistency was excellent in the current study (α = 0.94).

##### Hopkins symptom checklist – 25 (HSCL-25)

 Hopkins Symptom Checklist measures symptoms of depression and anxiety [35]. The abbreviated Swedish 25-item version (HSCL-25) was used [36]. Items are rated on a four-point Likert scale ranging from 1 (“not at all”), to 4 (“very much”). The scale comprises two subscales: a 15-item depression subscale, and a 10-item anxiety subscale. Mean scores are calculated for each subscale, with scores ranging from 1 to 4. A global score is calculated as the mean across all items. Higher scores indicate greater levels of depressive and anxiety symptoms. The original scale has demonstrated good internal consistency [35]. Nettelbladt et al., [36] suggested a cut-off score of $\:\ge\:\:$ 1.75 as indicative of psychopathology. Internal consistency was excellent in the current study (α = 0.94).

##### Deliberate self-harm inventory (DSHI)

The Deliberate Self-Harm Inventory (DSHI) [37] is a self-report questionnaire assessing the frequency of NSSI during the past six months. In the current study, the abbreviated Swedish 9-item version (DSHI-9) was used [38, 39]. The frequency of each NSSI method is rated on a scale from 0 (“never”) to 6 (“more than five times”). Items are summed up to produce a total score ranging from 0 to 54, with higher score indicating a higher frequency of NSSI. Two additional items assess whether NSSI has resulted in a need for hospitilisation and/or medical care, as well as when the individual last engaged in NSSI. Previous research has demonstrated acceptable to good internal consistency (α = 0.66-0.85) and variable test-retest reliability, ranging from *r* = .85 (*p* = .001) at 44 days to *r* = .27 (*p* = .08) at 126 days [38].

##### Eating disorder examination (EDE-Q)

The Eating Disorder Examination Questionnaire (EDE-Q) is a 36-item self-report questionnaire assessing ED symptoms during the past 28 days [40]. Items are assessed on a 7-point Likert scale, yielding a global score and four subscales: Eating Concern, Shape Concern, Weight Concern and Restraint. Mean scores are calculated for each subscale, while the global score is calculated as the mean of the four subscale scores. Higher scores indicate greater severity of ED symptoms. In addition, the EDE-Q assesses the frequency of ED-related behaviours (e.g. binge eating and compensatory behaviours). These items are not included in the calculation of the global or subscale scores. Previously, a global score of$\:\ge\:$ 4.0 was considered indicative of clinically significant ED symptoms [41, 42]. However, Swedish normative data suggest that this cutoff may be too high 17, and thus$\:\ge\:$ 2.76 have been used in recent Swedish research [43]. The scale has demonstrated acceptable to excellent internal consistency (ranging from 0.70 to 0.93), acceptable to excellent test-retest reliability across the four subscales, and has been shown to differentiate individuals with and without ED [1]. The Swedish version of the EDE-Q was used, for which normative data are available 17. 

### Statistics

IBM SPSS Statistics version 28.0.1.0 was used for all statistical analyses, except for the latent class analysis, which was conducted in R studio version 4.4.2.

First, one-way analyses of variance (ANOVAs) were conducted to examine differences in the CIA global score and subscale scores across ED diagnostic groups, addressing the first study aim regarding psychosocial functioning across EDs. Eta squared was used as a measure of effect size [44]. Tukey’s multiple comparison post hoc test was used to identify differences between ED groups, and Hedges g was calculated as a measure of effect size [45].

Second, latent class analysis was conducted using the R package poLCA, version 1.6.0.1, to identify subgroups based on DERS-16 scores. Models specifying two to five latent classes were tested. The optimal number of classes was determined based on the − 2 Log Likelihood (-2LL), Bayesian Information Criterion (BIC) and Akaike Information Criterion (AIC).

Generalised linear models (GLMs) were then used to examine associations between latent class membership and the theorised dependent variables. Clinical variables (depression and anxiety, NSSI (DSHI-9r)) and indicators of eating pathology (EDE-Q and CIA social impairment, respectively) served as observed outcomes. The CIA social impairment subscale was selected instead of the global CIA score due to indications of multicollinearity between the global CIA score and EDE-Q scores (*r* = .739) In comparison, the correlation between CIA social impairment and EDE-Q was lower (*r =* .660*).* The social impairment subscale includes several behavioural items (e.g. hindered from socialising and experiencing impaired work performance). A normal distribution with an identity link was used for social impairment, EDE-Q, and HSCL depression and anxiety scores. A binomial distribution with a logit link was used for binge eating (EDE-Q item 18), vomiting (EDE-Q item 22), and other compensatory behaviour (EDE-Q items 24, 26 and 28). These variables were dichotomised, with 0 indicating no occurrence and 1 indicating any occurrence. A negative binomial distribution with a log link was specified for DSHI-9r scores to account for the floor effect observed in self-reported NSSI. Model selection was based on Akaike’s Information Criterion (AIC) and Bayesian Information Criterion (BIC), allowing evaluation of both model fit and parsimony.

For the third study aim, a directed acyclic graph was used to determine which variables should be included as covariates in the regression model. Given the high correlation between EDE-Q and CIA global scores in the current study (*r* = .739, *p* < .001), as well as correlations reported in previous studies (*r* = .72; [46] and $\:\rho\:\:$= 0.71–89; [13, 47, 48]), diagnostic group (ANr, ANbp, BN, OSFED AN, OSFED BN) was selected instead of self-reported ED symptoms measured by the EDE-Q, due to the risk of multicollinearity. Hierarchical multiple linear regression was used to examine whether emotion dysregulation (DERS-16) was associated with psychosocial functioning (CIA global score), controlling for depressive and anxiety symptoms (HSCL-25), ED diagnosis, and age. The assumptions of multiple linear regression were assessed using visual inspection, quantile-quantile plots, plotting standardised residuals against standardised predicted values, and bivariate correlations. The data were considered suitable for multiple regression analysis, apart from the mentioned risk for multicollinearity.

Missing data were handled using mean imputation for the global scores of the CIA, DERS-16, and HSCL-25 (< 0.02% items across scales). Mean imputation was performed only when fewer than 15% of items were missing on a given global scale; otherwise, the participant was excluded from the relevant analysis. Imputed scores were rounded to the closest integer to reflect the less severe level of symptoms or impairment.

## Results

### Psychosocial functioning across EDs

The first aim was to descriptively investigate psychosocial functioning across EDs. Mean CIA global and subscale scores were compared across ED groups. A significant difference was found in the global CIA score (*F*(4, 183) = 3.92, *p* < .004, *n*^*2*^ = 0.08). Post hoc analyses were carried out, see Table 2. ANr and OSFED BN had the lowest mean global CIA scores (i.e. the lowest levels of psychosocial impairment). BN and OSFED AN had the highest mean global CIA scores (i.e. the highest levels of global psychosocial impairment). Effect sizes were very small to moderate; ANr vs. OSFED BN (*g =* 0.10) and BN vs. OSFED AN (*g* = 0.14), ANr vs. ANbp (*g* = 0.30), ANbp vs. BN (*g* = 0.24), ANbp vs. OSFED AN (*g* = 0.35), ANbp vs. OSFED BN (*g* = 0.38), ANr vs. BN (*g* = 0.59), ANr vs. OSFED AN (*g* = 0.72), BN vs. OSFED BN (*g* = 0.67), OSFED AN vs. OSFED BN(*g* = 0.77).

**Table 2:** Psychosocial functioning across EDs, represented in M (SD) CIA scores. *n* = 193

	ANr *n* = 30	ANbp *n* = 32	BN *n* = 74	OSFED AN *n* = 25	OSFED BN *n* = 32	Post hoc tests Tukey’s HSD
CIA global score^a^	29.76 (7.91)	32.45 (9.76)	34.58 (8.32)	35.76 (8.84)	28.90 (9.04)	BN> OSFED BN*, OSFED AN> OSFED BN*
CIA subscale Personal impairment^a^	2.32 (0.55)	2.48 (0.63)	2.66 (0.41)	2.61 (0.59)	2.33 (0.69)	BN > ANr*
CIA subscale Social impairment^a^	1.61 (0.71)	1.69 (0.81)	1.86 (0.77)	2.04 (0.77)	1.54 (0.70)	
CIA subscale Cognitive impairment^a^	1.57 (0.64)	1.83 (0.71)	1.87 (0.77)	1.98 (0.56)	1.44 (0.66)	BN> OSFED BN*, OSFED AN> OSFED BN*

Note. ANr: Anorexia nervosa restrictive, ANbp: Anorexia nervosa binge-eating/purging, OSFED AN: atypical anorexia nervosa, BN: Bulimia nervosa, OSFED BN: Otherwise specified feeding or eating disorder binge-eating/purging. *p < .05. a: ANr, ANbp, and OSFED BN had one missing each. The BN group had two missing.

Regarding the subscales, significant differences were found for CIA personal impairment (*F*(4, 183) = 3.256, *p* = .013, *n*^*2*^ = 0.066), and CIA cognitive impairment (*F*(4, 183) = 3.35, *p* = .011, *n*^*2*^ = 0.07). No significant difference was found for CIA social impairment (*F*(4, 183) = 2.16, *p* = .075, *n*^*2*^ = 0.05). See Table 2 for further details.

### Classifying patients based on their emotion regulation profiles

The second aim was to examine whether individuals with EDs form distinct subgroups based on emotion dysregulation. Latent class analysis was conducted to determine whether subgroups could be found. Table 3 summarises the fit indices for all models estimated in the latent class analysis.

**Table 3:** Estimated models with fit measures

				Proportion of participants in class				
Classes	-2LL	BIC	AIC	Class1	Class2	Class3	Class4	Class5
1	-4677.297	9690.403	9483.594	1				
2	-4083.377	8843.621	8424.754	0.447	0.553			
3	-3861.981	8741.885	8111.963	0.275	0.389	0.321		
4	-3743.987	8846.954	8005.974	0.247	0.216	0.263	0.274	
5	-3672.978	9045.991	7993.955	0.189	0.174	0.137	0.205	0.295

Note. Lower values of the Bayesian information criteria (BIC) and Aikaike’s information criteria (AIC) indicate better fitting models

**Fig. 2:**
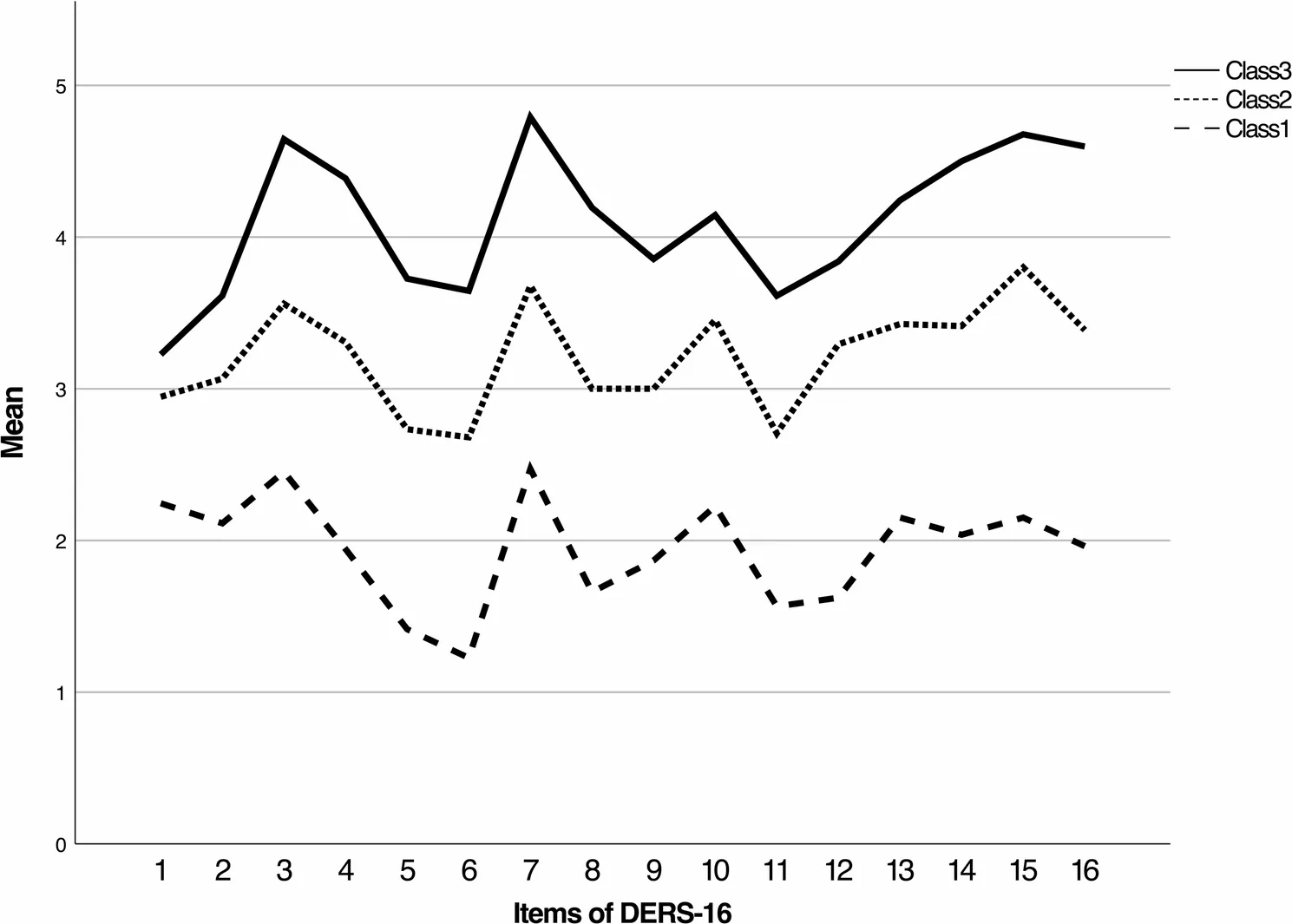
Latent classes on items of Difficulties in Emotion Regulation Strategies 16

The three-class model was selected as the best-fitting model based on the BIC, which adjusts for degrees of freedom and thereby reduces the likelihood of overfitting. Participants were relatively evenly distributed across the three classes: *n =* 53 (27.5%) in Class 1, *n* = 75 (38.9%) in Class 2, *n* = 62 (32.1%) in Class 3. Figure 2 provides graphical representation of the three classes. Class 1 represents low DERS scores, Class 2 intermediate DERS scores and Class 3 high DERS scores.

### Clinical characteristics associated with class membership

As the findings from the latent class analysis indicated that there are three subgroups of emotion dysregulation in the ED population, associations between latent class membership and clinical outcomes were explored. Thus, generalised linear models were used to examine this, summarised in Table 4. Compared with Class 1 (low DERS scores), Class 2 (intermediate DERS scores) showed higher levels of depressive and anxiety symptoms and NSSI. Compared with Class 2 (intermediate DERS scores), Class 3 (high DERS scores) showed higher EDE-Q scores and higher levels of depressive and anxiety symptoms but was less likely to engage in other compensatory behaviour (i.e., laxative or diuretic use, or excessive exercise). Comparisons between Class 3 and Class 1 showed significant differences across all outcomes except for vomiting and binge eating.

**Table 4:** Generalised linear models – class membership and clinical outcomes for the three-class solution

	Mean (standard error)					
Continuous outcomes		Class 1	Class 2	Class 3	Overall$\:{\chi\:}^{-2}$	*p* value
EDE-Q		3.91 (0.14)	4.10 (0.12)	4.51 (0.13)	10.22^b, c^	**0.006**
HSCL-25 Depression		2.10 (0.08)	2.63 (0.07)	3.0 (0.07)	60.01 ^a, b, c^	**< 0.001**
HSCL-25 Anxiety		2.04 (0.08)	2.38 (0.07)	2.86 (0.07)	51.58 ^a, b, c^	**< 0.001**
Non-suicidal self-injury DSHI-9r		1.75 (0.30)	4.88 (0.62)	5.69 (0.78)	29.49^a, b^	**< 0.001**
	Probability (standard error)					
Binary outcomes		Class 1	Class 2	Class 3	Overall$\:{\chi\:}^{-2}$	*p* value
Vomiting		0.52 (0.07)	0.39 (0.06)	0.42 (0.06)	2.29	0.319
Binge eating		0.44 (0.07)	0.41 (0.06)	0.31 (0.06)	2.50	0.286
Other compensatory behaviour		0.56 (0.7)	0.52 (0.06)	0.34 (0.06)	6.74 ^b, c^	**0.034**

Note. Regarding the binary outcomes, the probabilities of engagement in the given behaviour are reported; *df* = 2 for all outcomes; Other compensatory behaviour = Use of laxatives, diuretics, or excessive training. ^a^Class 1 versus 2, *p* < .05. ^b^Class 1 versus 3, *p* < .05. ^c^Class 2 versus 3, *p* < .05.

### The relationship between emotion regulation and psychosocial functioning in EDs

The third aim was to examine the association between emotion dysregulation and psychosocial impairment. In a simple correlation, DERS-16 and CIA global score had a small positive correlation (*r = .*26, *p* = < 0.001). A hierarchical multiple linear regression was conducted to test the association between DERS-16 and CIA global scores. First, Model 1 included age, and was not statistically significant (*F*(1, 184) = 2.99, *p* = .086, *R*^*2*^ = 0.016). Second, Model 2 included ED diagnostic groups, as well as the HSCL-25 depression and anxiety subscales. Model 2 was statistically significant (*F*(7, 178) = 13.19, *p* < .001, *R*^*2*^ = 0.342), contributing with additionally 32.6% unique variance explained. Third, Model 3 added DERS-16, and was statistically significant (*F*(8, 177) = 12.05, *p* = < 0.001, *R*^*2*^ = 0.353), see Table 5 for further details. However, Model 3 did not explain a significant increase in variance compared with Model 2 (*F* change, *p* = .085). Thus, after controlling for age, diagnostic group, and symptoms of depression and anxiety, emotion dysregulation did not account for significant additional variance in psychosocial impairment. This was contrary to the hypothesis, and Model 2 was therefore retained as the final model. In Model 2, an OSFED BN diagnosis was significantly associated with lower CIA score, while higher HSCL-25 depression subscale scores were significantly associated with higher levels of psychosocial impairment, see Table 5. These findings suggest that the association between emotion dysregulation and psychosocial impairment did not explain significant unique variance beyond that explained by depressive symptoms and other covariates.

**Table 5:** Linear model of predictors of CIA global scale (*n* = 186)

	B (SE B)	ß	*p*
Model 1			
Constant	36.92 (2.52)		**< 0.001**
Age	− 0.17 (0.10)	-1.26	0.086
Model 2			
Constant	18.81 (3.64)		**< 0.001**
Age	− 0.10 (0.08)	− 0.08	0.235
ANr	-3.14 (1.69)	− 0.13	0.064
ANbp	-2.48 (1.63)	− 0.10	0.129
OSFED AN	− 0.54 (1.78)	− 0.02	0.760
OSFED BN	-4.56 (1.64)	− 0.19	**0.006**
HSCL-25 Depression	5.29 (1.24)	0.40	**< 0.001**
HSCL-25 Anxiety	1.80 (1.31)	0.13	0.170
Model 3			
Constant	20.74 (3.79)		**< 0.001**
Age	− 0.12 (0.08)	− 0.09	0.157
ANr	-3.53 (1.69)	− 0.14	**0.038**
ANbp	-2.76 (1.63)	− 0.11	0.092
OSFED AN	-1.00 (1.79)	− 0.04	0.577
OSFED BN	-4.77 (1.64)	− 0.20	**0.004**
HSCL-25 Depression	5.99 (1.29)	0.46	**< 0.001**
HSCL-25 Anxiety	2.19 (1.32)	0.16	0.098
DERS-16	− 0.08 (0.05)	− 0.13	0.085

Note. Model 1: *R*^*2*^ = 0.016, *p* = .086; Model 2:*R*^*2*^ = 0.326, *p* = < 0.001; Model 3:*R*^*2*^ = 0.011, *p* = .085. ANr: Anorexia nervosa restrictive, ANbp: Anorexia nervosa binge-eating/purging, OSFED AN: atypical anorexia nervosa, BN: Bulimia nervosa, OSFED BN: Otherwise specified feeding or eating disorder bulimia nervosa and purging disorder

Additionally, hierarchical multiple linear regression was conducted to test the association between emotion dysregulation and psychosocial impairment assessed by the CIA subscales. Regarding the CIA social and personal impairment subscales, the results were essentially the same as the global score. Thus, these are not reported in detail. The CIA cognitive impairment subscale showed partly differing results. Both HSCL-25 depression and anxiety subscales had significant beta weights in the final regression model.

## Discussion

In the current study, we explored psychosocial impairment, subgroups of emotion dysregulation, and the association between emotion dysregulation and psychosocial impairment among individuals with ED. First, our findings indicated differences in levels of psychosocial impairment across EDs. The BN and OSFED AN groups self-reported higher levels of psychosocial impairment, compared to the OSFED BN group. Second, three subgroups characterised by low, intermediate and high emotion dysregulation were identified and linked to distinct clinical profiles. Third, emotion dysregulation was not significantly associated with psychosocial impairment after controlling for depressive symptoms.

### Psychosocial functioning across EDs

Regarding the first aim, the findings suggest that the BN and OSFED AN groups are more impaired by their ED than the OSFED BN group, as indicated by self-ratings. This is consistent with patterns observed in sick-leave rates (BN: 19.2%, OSFED AN 32.0%), see Supplementary Table6. This similarly suggests a higher occupational impairment. One possible explanation for the lower levels of impairment observed in the OSFED BN group is that these individuals may have lower symptom frequency or severity than those meeting the full criteria for BN. This may result in less disruption to daily functioning. This pattern may be more characteristic of bulimic presentations and may not extend to anorexic presentations. However, previous findings indicate that OSFED show similar ED psychopathology to threshold EDs [49].

Previous research has yielded mixed findings regarding differences in impairment across EDs. Partly in line with our results, individuals with BN and AN have been reported to be more impaired compared to EDNOS 17. In contrast, other research has showed no difference in impairment across ED diagnoses [14–16]. These differences in findings indicate that the relationship between psychosocial functioning and EDs may be more complex than the current study examined. To better understand these divergent results, it may therefore be important to consider factors accounting for the variation across studies. One such factor is depression rather than ED diagnosis itself. Depression has been linked to psychosocial impairment in adolescents with EDs [50]. Future research should therefore examine whether differences in psychosocial impairment across ED diagnoses are better explained by depressive symptoms or other transdiagnostic factors, in adults. This may also have clinical implications, as previous research suggests poorer psychosocial functioning predicts relapse in BN [51], indicating that psychosocial functioning may represent an important treatment target.

Beyond the main finding, an additional pattern emerged in relation to psychosocial impairment. The ANr group self-reported the second lowest level of psychosocial impairment among the ED groups. Although the post hoc analyses did not indicate statistically significant differences, the effect sizes suggested moderate differences between the ANr group and both the BN and OSFED AN groups. However, these findings should be interpreted with caution. There may be no true difference in psychosocial impairment between the ANr and other diagnostic groups. Alternatively, relatively small sample size may have limited statistical power to detect group differences. Interestingly, self-reported low levels of psychosocial impairment appear inconsistent with demographic characteristics. The ANr group has the lowest BMI (*M* = 16.7, *SD* = 1.1), highest proportion of participants who were single (69.0%), and second-highest proportion with no current occupation (unemployed and sick-leave: 24.1%). See Supplementary Table 6 for further details. These findings contrast with the self-reported impairment on the CIA social subscale, which assesses impairment in work performance and social relationships. Some research indicates that the group underreport their impairment, e.g. up to a third of individuals hospitalised with AN scored within the normal range on EDE or EDE-Q [52]. Other research suggests that the AN group show a marked perseverance in remaining active and working despite starvation [53], as well as having higher grades and educational attainment [54]. This may possibly be tied to traits of overcontrol, such as inhibition and perfectionism [31].

The demographic characteristics of the current sample were compared with those reported in previous clinical and epidemiological studies of individuals with EDs. A Swedish registry study including patients receiving specialised ED treatment during 2012–2016 reported somewhat different distribution of diagnostic groups [55]. Among adults, AN accounted for 20% of patients, while BN accounted for 32%, compared to 32% and 38% respectively, in the current sample. Eating Disorders Not Otherwise Specified accounted for 37% in the registry study, whereas OSFED AN and OSFED BN together accounted for 30% in the current sample. Men comprised 4.6% of patients, compared to 4% (the excluded *n* = 8). Compared with the 12-month prevalence estimates from a nationally representative, population-based epidemiological study of US adults, the BN group in the current study had a lower proportion of individuals with primary-level education. In the AN group, higher educational attainment was more common in the epidemiological sample than in the current sample [56]. In a Swedish register-based study, 80% of participants had completed at least secondary education [25], compared with 85% in the current sample. Moreover, the age of the current sample appeared to be broadly consistent with that reported by Udo and Grilo [56], with ANr (*M* = 24.7) and ANbp (*M* = 24.5) being most commonly represented in the 18–29 age range. The BN group had a mean age of 27.3 years, with BN being most common in both the 18–29 and 30–44 age ranges in Udo and Grilo [56]. See Supplementary Table 6 for further details. Similarly, a Swedish register-based study found the mean age of diagnosis to be younger for AN (*M* = 20.8) than for BN (*M* = 23.6) [57]. Although the mean age in our sample was somewhat higher, the same pattern was observed, consistent with findings from Udo and Grilo [56]. However, direct comparisons are limited as age at diagnosis and age at clinical assessment reflect different time points; participants may have had a diagnosis before they were clinically assessed in our study.

### Classifying patients based on their emotion regulation profiles

Regarding the second aim, a three-class solution provided the best fit for the data, indicating the presence of three subgroups characterised by different levels of emotion dysregulation. This interpretation is further supported by the class-specific mean scores across DERS-16 items, see Fig. 2. There is a clear separation between the three classes, with no apparent overlap in their mean levels of emotion dysregulation. Taken together, these findings suggest meaningful heterogeneity in emotion dysregulation among individuals with EDs, ranging from relatively low to substantially elevated levels of impairment, i.e. a quantitative difference. Further, the three-class model was validated against clinical variables. Symptoms of eating pathology, depression, anxiety, as well as NSSI all followed a similar pattern; the higher emotion dysregulation, the more severe symptoms and more frequent NSSI.

In contrast, other compensatory behaviours (use of laxatives, diuretics or excessive training) were significantly more prevalent in Class 1 (low DERS scores) and Class 2 (intermediate DERS scores), compared to Class 3 (high DERS scores). Largely, the current findings are in line with those reported by Nordgren et al., [25]. Both studies have found a three-class model, and both have validated against clinical variables. The three class models both show association between higher emotions dysregulation and ED severity, elevated levels of symptoms of depression and anxiety, and NSSI. However, some differences were observed between the two studies. The current study found the reversed pattern between EDE-Q other compensatory behaviours (i.e. more prevalent in classes with lower levels of emotion dysregulation), whereas Nordgren et al., [25] found no significant class difference. In addition, no difference were found between classes in prevalence of vomiting and binge eating in the current study, whereas Nordgren et al., [25] reported significant differences between classes for both behaviours. The most notable difference between the two studies is that Nordgren et al., [25] identified qualitatively distinct classes across clinical variables, whereas the current study suggests that the class differences are primarily quantitative rather than qualitative. The current study adds to the literature suggesting that emotion regulation may be a transdiagnostic treatment target to consider (e.g. [58], [22], [23]

Although the current focused on subgrouping patients according to their emotion regulation profiles, it did not account for the different styles of emotion regulation. ANr and OSFED AN have previously been linked to an overcontrolled style of emotion regulation characterised by excessive rigidity, inhibition and perfectionism [31]. This indicates that there may be multiple emotion regulation deficits in the ED population, which is not captured by the current findings.

### The relationship between emotion regulation and psychosocial functioning in EDs

The third aim was to examine the association between emotion dysregulation and psychosocial impairment. Contrary to the hypothesis, emotion dysregulation was not significantly associated to psychosocial impairment, after controlling for depressive symptoms. In addition, OSFED BN was found to be associated with lower psychosocial impairment, as already indicated by findings reported in the first aim. These findings indicate that emotion dysregulation does not explain unique variance beyond that of depressive symptoms. This may suggest that depressive symptoms are more important in understanding psychosocial impairment among individuals with ED, than emotion dysregulation. Previous findings show that EDs and depression are risk factors for each other, i.e. suggesting a bi-directional pathway between the two [59]. One potential clinical implication may be that symptoms of depression can be an important treatment target to improve psychosocial functioning in the ED patient group.

Emotion dysregulation and psychosocial functioning may be differentially associated across ED diagnoses, specifically anorexic and bulimic presentations. The lack of significant association may reflect compensatory behaviours that help maintain occupational functioning, particularly in AN, such as regulating emotions through starvation [60]. This may help explain the seemingly paradoxical finding that AN does not result in greater functional impairment than OSFED AN, despite a higher symptom burden. In contrast, emotion dysregulation may be more closely linked to behaviours such as purging and NSSI, which are more common in BN and ANbp [61] and may serve an emotion regulatory function [62].

To our knowledge, extant research has not examined the association between emotion dysregulation and self-assessed psychosocial impairment among individuals with EDs. However, the literature suggests that emotion dysregulation is associated with poorer psychosocial functioning in several psychiatric conditions (e.g. [27], [28], [29], [26]. The current study did not include clinician-rated assessments of psychosocial impairment. Future research may therefore benefit from examining the association between clinician-rated impairment and emotion dysregulation among individuals with EDs.

### Limitations

Several important limitations of the current study warrant consideration. One weakness of the study is not including a clinician-rated assessment of psychosocial impairment, e.g. global assessment of functioning (GAF)^9^ , in combination with the included self-assessed psychosocial impairment scale CIA. Combining the two would have allowed a comparison between self-assessment and clinician-rated impairment, as well as the different definitions of psychosocial impairment. The CIA assesses ED-specific psychosocial impairment (i.e. the effect of ED symptoms on the personal, cognitive and social domains) [13]. The GAF assesses psychological, social and occupational impairment [9]. A comparison between the two and how they may relate to emotion dysregulation and clinical variable would have been valuable. Especially considering that the AN group may have a tendency to underreport [52].

A further consideration is the relatively small diagnostic groups. Small group sizes may have reduced statistical power, increasing the risk of Type II errors. Thus, non-significant findings, particularly those between the diagnostic groups should be interpreted with caution (e.g. see the discussion regarding ANr above).

Finally, the representativity of the sample may represent a limitation. The current study included neither men and non-binary individuals, nor all eating disorders (e.g. binge eating disorder). Hence, the generalisability of the findings to the whole ED population becomes limited. Men and non-binary individuals were scarce in the current sample and were therefore excluded from the analyses. The sample was however relatively representative of the proportion of individuals with EDs who access ED treatment, i.e. the majority of ED patients are women. Men have lower prevalence of EDs (0.3–0.7%) compared to women (AN: 1–4%, BN: 1–2%), and only about one third of individuals with EDs access healthcare [63]. In addition, men’s help-seeking behaviour is hampered due to notions about ED being exclusive to women [64]. Thus, a substantially larger sample size than that of the current study would be required to capture a sufficiently large number of males with EDs.

## Conclusions

Our findings suggest that patients with BN and OSFED AN have the highest levels of self-assessed psychosocial impairment, whereas those with OSFED BN and ANr have the lowest. Three subgroups characterised by different levels of emotion dysregulation were identified and associated with distinct clinical profiles. Emotion dysregulation was not significantly associated with psychosocial functioning, when controlling for depressive symptoms. Possible implications may be that depression can be a relevant treatment target among individuals with EDs. In addition, emotion dysregulation may represent an important treatment target for a subgroup of patients with EDs. Future research may further examine emotion dysregulation and its association to psychosocial impairment, especially clinician-rated impairment. Another focus may be different emotion regulation deficits (e.g. overcontrolled), as well as exploring whether a subgroup of patients may benefit from focusing on emotion dysregulation in treatment.

## Supplementary information

Below is the link to the electronic supplementary material.

Supplementary Material 1

## Data Availability

The dataset used in the current study is not publicly available due to restrictions in Swedish law but may be made available from the corresponding author upon reasonable request.

## Abbreviations

ANbp: Anorexia Nervosa binge eating/purging type
ANr: Anorexia Nervosa restricting type
BMI: Body Max Index
BN: Bulimia Nervosa
CIA: Clinical Impairment Assessment questionnaire
DERS-16: Difficulties in Emotion Regulation Scale (brief version)
DSHI-9: Deliberate Self-Harm Inventory – 9
ED: Eating Disorder
EDE: Eating Disorder Examination interview
EDE-Q: Eating Disorder Examination Questionnaire
EDNOS: Eating Disorders Not Otherwise Specified
HSCL-25: Hopkins Symptom Checklist – 25
NSSI: Non-Suicidal Self-Injury
OSFED AN: Other Specified Feeding or Eating Disorder Atypical Anorexia Nervosa
OSFED BN: Other Specified Feeding or Eating Disorder Bulimia Nervosa and Purging Disorder
