# Supplementary material for: Impaired psychosocial functioning and emotion regulation difficulties in eating disorders: a cross-sectional study
Source: J Eat Disord. 2026 Oct 22;14:249. doi: 10.1186/s40337-026-01803-7 (PMC13692984; doi:10.1186/s40337-026-01803-7)
Supplement: Supplementary file 1 [file 40337_2026_1803_MOESM1_ESM.docx]

Supplementary material

| **Supplementary Table 6**  *Demographic characteristics of study participants by diagnostic group n = 193* | | | | | | |
| --- | --- | --- | --- | --- | --- | --- |
|  | ANr  *n* = 30 | ANbp  *n* = 32 | BN  *n* = 74 | OSFED AN  *n* = 25 | OSFED BN  *n* = 32 | All participants |
| Mean age (*SD*) | 24.7 (5.0) | 24.5 (6.6) | 27.3 (7.8) | 23.0 (3.8) | 24.4 (6.7) | 25.4 (6.8) |
| Mean BMI (*SD*)^a^ | 16.7 (1.2) | 17.0 (1.1) | 24.5 (4.6) | 20.1 (1.9) | 23.9 (4.4) | 21.4 (4.8) |
| Marital status^b^ |  |  |  |  |  |  |
| Partner | 7 (24.1%) | 10 (32.3%) | 25 (33.8%) | 11 (44.0%) | 12 (38.7%) | 65 (33.7%) |
| Single | 20 (69.0%) | 19 (61.3%) | 46 (62.2%) | 13 (52.0%) | 19 (61.3%) | 117 (60.6%) |
| Other (e.g. living  with parents) | 2 (6.9%) | 2 (6.5%) | 3 (4.1%) | 1 (4.0%) | 0 (0.0%) | 8 (4.1%) |
| Highest level of education^c^ |  |  |  |  |  |  |
| Primary school | 5 (17.2%) | 6 (18.8%) | 9 (12.2%) | 4 (16.0%) | 4 (12.5%) | 28 (14.5%) |
| Secondary  school | 14 (48.3%) | 16 (50.0%) | 33 (44.6%) | 10 (40.0%) | 20 (62.5%) | 93 (48.2%) |
| Higher education | 10 (34.5%) | 10 (31.3%) | 32 (43.2%) | 11 (44.0%) | 8 (25.0%) | 71 (36.8%) |
| Main occupation^d^ |  |  |  |  |  |  |
| Paid work | 8 (27.6%) | 10 (31.3%) | 32 (43.8%) | 6 (24.0%) | 12 (37.5%) | 68 (35.3%) |
| Student | 13 (44.8%) | 16 (50.0%) | 26 (35.6%) | 9 (36.0%) | 15 (46.9%) | 79 (40.9%) |
| Unemployed | 2 (6.9%) | 0 (0.0%) | 0 (0.0%) | 2 (8.0%) | 0 (0.0%) | 4 (2.1%) |
| Sick leave | 5 (17.2%) | 5 (15.6%) | 14 (19.2%) | 8 (32.0%) | 4 (12.5%) | 36(18.7%) |
| Other (e.g.  parental leave,  internship) | 1 (3.4%) | 1 (3.1%) | 1 (1.4%) | 0 (0.0%) | 1 (3.1%) | 4 (2.0%) |
| *Note.* ANr: Anorexia nervosa restrictive. ANbp: Anorexia nervosa binge eating/purging. BN: Bulimia Nervosa. OSFED AN: Atypical anorexia nervosa. OSFED BN: Other specified feeding or eating disorder.  a: BN, OSFED AN, and OSFED BN had one missing each on BMI  b: ANr, ANbp, and OSFED BN had one missing each on marital status  c: ANr had one missing on highest level of education  d: ANr, and BN had one missing each on main occupation | | | | | | |
